# Machine Learning Approaches for the Prediction of Displaced Abomasum in Dairy Cows Using a Highly Imbalanced Dataset

**DOI:** 10.3390/ani15131833

**Published:** 2025-06-20

**Authors:** Zeinab Asgari, Ali Sadeghi-Sefidmazgi, Abbas Pakdel, Saleh Shahinfar

**Affiliations:** 1 Department of Animal Sciences, College of Agriculture, Isfahan University of Technology, Isfahan 84156-83111, Iran; zeinab.asgari98@ag.iut.ac.ir (Z.A.); pakdel@iut.ac.ir (A.P.); 2 Department of Animal Science, University of Tehran, Karaj 3158711167-4111, Iran; sadeghism@ut.ac.ir; 3 Department of Health and Aged Care, Canberra, ACT 2606, Australia

**Keywords:** health, welfare, artificial intelligence, gradient boosting model, imbalanced data

## Abstract

Displaced abomasum (DA) is a digestive disorder that causes severe economic losses through the reduction in milk yield and early culling of cows. The predictive potential of DA-susceptible cases is of great importance to reduce economic losses. This study aimed for early prediction of DA. However, identifying cows at risk of DA can be difficult because DA is a complex trait and its incidence is low. For this purpose, in this study, the ability of five machine learning algorithms, namely Logistic Regression (LR), Naïve Bayes (NB), Decision Tree, Random Forest (RF) and Gradient Boosting Machines (GBM), to predict cases of DA was investigated. For these predictions, 20 herd–cow-specific features and sire genetic information from 7 Holstein dairy herds that calved between 2010 and 2020 were available. Model performance metrics indicated that GBM and RF algorithms outperformed the others in predicting DA with F2 measures of 0.32. The true positive rate in the RF was the highest compared to other methods at 0.75, followed by GBM at 0.70. Given the highly imbalanced data, this study showed the potential in forecasting cases susceptible to DA. This prediction tool can aid dairy farmers in making preventative management decisions by identifying cows susceptible to DA.

## Simple Summary

Displaced abomasum (DA) is one of the costliest health problems as well as a welfare concern in dairy cows. Therefore, the predictive potential of DA-susceptible cases is of great importance to reduce economic losses. Hence, this study aimed for early prediction of DA by using five machine learning algorithms, namely Logistic Regression (LR), Naïve Bayes (NB), Decision Tree, Random Forest (RF) and Gradient Boosting Machines (GBM), to predict cases of DA. Model performance metrics indicated that among the algorithms considered in this study, RF and GBM were significantly better in terms of F2 (0.32) and TPR (0.75 and 0.70, respectively). Therefore, given the highly unbalanced data and that DA is a complex feature, machine learning (ML) methods were shown to be promising for predicting cases susceptible to DA at the herd level. This prediction tool can aid dairy farmers in making preventative management decisions by identifying cows susceptible to DA.

## 1. Introduction

Displaced abomasum is defined as abomasum mislocated from its normal position to either the left or right side [1] and is considered the most common surgical gastrointestinal disorder in dairy cows [2]. Approximately 30–50% of dairy cows suffer metabolic and infectious diseases after calving [3] and the annual incidence of DA has been reported to range between 0.05% and 6% [4]. DA is an important digestive condition that leads to great concern in terms of animal economics [5] and has a detrimental impact on animal welfare due to disease occurrence, health outcomes and the experience of pain [6], which in turn can influence the profitability in the herd [7]. The direct economic impact of DA includes the costs of treatment, early culling, and the decrease in milk production [8], an increase in the interval of calving to first service [9,10]. Although ketosis is a well-recognized risk factor for left displacement abomasum (LDA), in some studies, the occurrence of some disorders such as ketosis following DA has been reported [11,12,13]. In this regard, it should be noted that sometimes DA may have occurred but had not yet been diagnosed. At this time, the abomasum is periodically filled with gas and fluid, and then emptied. Therefore, in cows with uncomplicated displacements, where the cow may go through periods of apparent well-being, ketosis can develop due to the reduction in feed consumption [14]. Direct and indirect cost per case of DA in the United States have been reported to be more than 700 USD [8]. Hence, the ability to predict cases susceptible to DA could allow for earlier intervention strategies.

DA multifactorial disease occurs mostly in the early postpartum period [10] and depends on the coordinated action of a set of predisposing factors [15,16]. According to previous reports, more than 50% of DA cases occur in the first postpartum period [9,17]. Some earlier studies have reported factors that are associated with the likelihood of DA occurrence [4,13,17,18]. The known non-genetic factors contributing to the incidence of DA include herd, year and season of calving, parity, reproductive and other metabolic disorders [17,18,19,20]. Also, DA is largely affected by nutrition management and concentration of various metabolites [21,22]. In addition to cow- and herd-level factors, numerous studies suggest that DA is a heritable trait [23,24], with an estimated heritability between 0.12 and 0.32 [23]. This means that up to 30% of the phenotypic variation in the DA at the population level can be explained by additive genetic variations. Also, a positive genetic correlation (0.45 ± 0.16) is reported for DA with other metabolic disorders such as ketosis [25].

To monitor the metabolic status of cows, measuring plasma metabolites and metabolic hormones has been suggested [3], but these metabolites and hormones are difficult to measure in the farm [26]. In recent years, animal studies have used machine learning methods to predict traits such as assessing disease impact in dairy cattle [7], assessing the impact of technology integration on cow health [27], the incidence of lameness [28], the incidence of neosporosis in cattle [29], ketosis [30], the incidence of retained placenta [31], and the metabolic status of dairy cows [26]. Machine learning algorithms can deal with inter-correlations [32] and analyze large sets of data, regardless of complexity, quickly and accurately [33]. Machine learning methods have previously been used to predict complex traits [28]; hence, according to the fact that DA is a multifactor trait, the present study was conducted with the aim to investigate machine learning method capabilities in predicting whether a cow is at high risk of DA during the lactation period.

## 2. Material and Methods

### 2.1. Data

The data used in this study consisted of health, reproduction, and production records extracted from backup files of Modiran (version 8.21.02.18), a Livestock Management Software, from 7 Holstein dairy farms located at 3 provinces of Iran (Esfahan, Chahar Mahaal en Bakhtiari, and Khorasan). Herds were selected based on their use of an accurate recording system (Livestock Management Software, Modiran 8.21.02.18), availability of data for DA, and other features required for analysis. All herds had comparably similar management routines in terms of veterinary services, vaccination and use of artificial insemination. The feed was as a balanced total mixed ration (similar ratio of forage to concentrate) and the free stall housing system. All herds on average contained ≥500 milking cows that were milked three times per day. Data extracted from the farm databases for each individual cow were: herd and cow identification, sire, lactation period, calving date, management factors, information on health and metabolic disorders and records of milk and milk compositions. Data were edited using R (version 4.1.2; [34]). Records with parity > 6, pregnancy period outside 240 to 298 days, age at first calving outside 578 to 1153 days and dry period length > 160 days were excluded from the analysis. Also records exceeding three standard deviations from the mean of milk yield, fat and protein and calf weight at birth were excluded. The number of excluded records and reasons for excluding them are presented in Table 1. After data cleaning from 93,273 available records, a total of 85,031 records were available following editing for statistical analysis; this included 56,368 multiparous cows and 28,663 primiparous cows.

Cases of DA, which included “all cases of DA” (i.e., left cases and right cases, 90% and 10%, respectively), are usually recorded as a binary trait (1 if a cow was affected by DA and 0 otherwise). Milk production traits including the monthly milk yield, fat and protein percentage (i.e., recorded on one day per month) before occurrence of DA for affected cows were considered (2841 cows). For cows with DA that had no prior milk record, the last record of the previous lactation was used (1394 cows). For unaffected cows, the first lactation record after calving was included in the dataset. Cows with DA and without a monthly record of milk were excluded from the analysis (582 cows). Other features used for predicting DA include parity (1, 2, …, 6), herd (h1, h2, …, h7), pregnancy length, calving season (spring, summer, fall and winter), dystocia and twinning (0 was normal partus, and 1 was abnormal partus), mastitis, ketosis, retained placenta and metritis during the current lactation and before the occurrence of DA, (0 for healthy cows and 1 for affected cows). It is necessary to explain that a positive test for the detection of the ketone bodies in blood (measurement of beta-hydroxybutyric acid (BHBA) in serum or plasma in the lab or using a ketone meter) combined with reduced feed intake was used to diagnose a cow with ketosis. The BHBA threshold used to diagnose subclinical ketosis was 1.2–2.9 mmol/L and for clinical ketosis was ≥3 mmol/L.

In addition to the herd–cow factors, sire EBVs (sire of the cow) obtained from the Alta Genetics website (https://bullsearch.altagenetics.com/us/BS/List, accessed on 1 February 2023) were also included in the dataset. This information includes the daughter pregnancy rate (PTA-DPR), productive life (PTA-PL), sire calving ease (PTA-SCE), sire stillbirth (PTA-SSB), daughter calving ease (PTA-DCE), daughter stillbirth (PTA-DSB), PTA of DA (PTA-DA), PTA of ketosis, PTA of somatic cell score (PTA-SCS), PTA of milk protein percentage and PTA of milk fat percentage. Therefore, they were considered as candidate predictor features for ML algorithms. A general description of predictor feature set available for predicting DA is provided in Table 2. Also, the frequency of each level of nominal features is shown the Table 3.

### 2.2. Machine Learning Methodology

In this study, five machine learning algorithms were used to predict the likelihood of DA incidence: Logistic Regression, Naïve Bayes, Decision Tree, Random Forest, and Gradient Boosting Machines. These models are described in detail in Shahinfar et al. [28,35].

After quality control and cleaning, the dataset contained 85,031 records (each record is a cow in a parity) of 47,686 Holstein cows calving between 2010 and 2020 and was divided into two sets with an 80:20 ratio as the training set and the hold-out testing set, respectively. Feature selection was performed to select the most informative features [36] only on the training dataset. After balancing the training set, 80% of the total training set was selected as the training set *i*, *i*∈{1:10}. Furthermore, 70% of the *i*th training set was randomly selected to be used for hyperparameter tuning via a nested grid search while internally tested on the remaining 30%. The best-performing hyperparameters in this step were used to train each ML algorithm within their corresponding *i*th training sets and were tested on the hold-out testing set at the end. The training algorithm is explained in detail in the next section.

#### 2.2.1. Feature Selection

**Figure 1:**
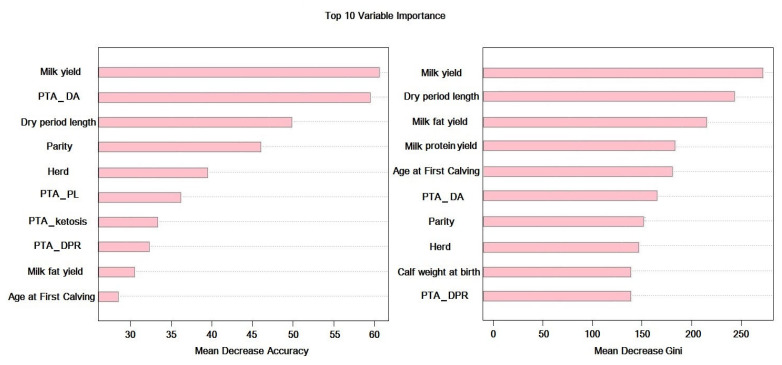
Variable importance plot of the top 10 predictor variables, according to the mean reduction in the Gini index and mean decrease in accuracy.

Feature selection was performed to discard uninformative features from the dataset [36]. Feature selection is extremely important in ML algorithms because it reduces computational costs, avoids overfitting [30], leads to the reduction in the distractive effect of noisy features [35], and by choosing the optimal subset of predictors, it promotes the efficiency of the model [37]. In the current study, Random Forest was performed to select the most effective set of features only on the training set. For this purpose, the mean reduction in the Gini index and the mean decrease in accuracy were used. Random Forest was performed with the number of trees to grow (500:1000) and the number of candidate variables randomly sampled in each split (4:5) in each iteration. The top 15 features for the mean decrease accuracy and the mean decrease in Gini index were selected and saved. Finally, the training process was conducted with the union of two feature sets selected. According to the results of trait importance and based on the intersection of both criteria considered in this study, milk yield, PTA_DA, dry period length, parity, milk fat yield, herd, AFC and PTA_DPR are the most important features in predicting DA (Figure 1). The selected features in the feature selection stage were highlighted in Table 2.

#### 2.2.2. Hyperparameter Tuning

The prediction models were developed using 10 repetitions as described in the next section. In each iteration, 80% of the training set was randomly selected and hyperparameter tuning was performed via grid search. Tuned hyperparameters were the number of trees to grow (100:500) and the number of candidate variables randomly sampled in each split (4:10) for RF. The Laplace smoothing coefficient (1:10) and the smallest allowed node size (10:100) were tuned for Naïve Bayes and Decision Tree, respectively. For GBM, the variable interaction levels (1:3) and the learning rate of 0.01, the number of trees (1000:1500), the fraction of the training set observations for the next tree (0.80 and 1), and the minimum number of observations in the terminal nodes of a tree (20 and 25) were tuned.

#### 2.2.3. Analysis

**Figure 2:**
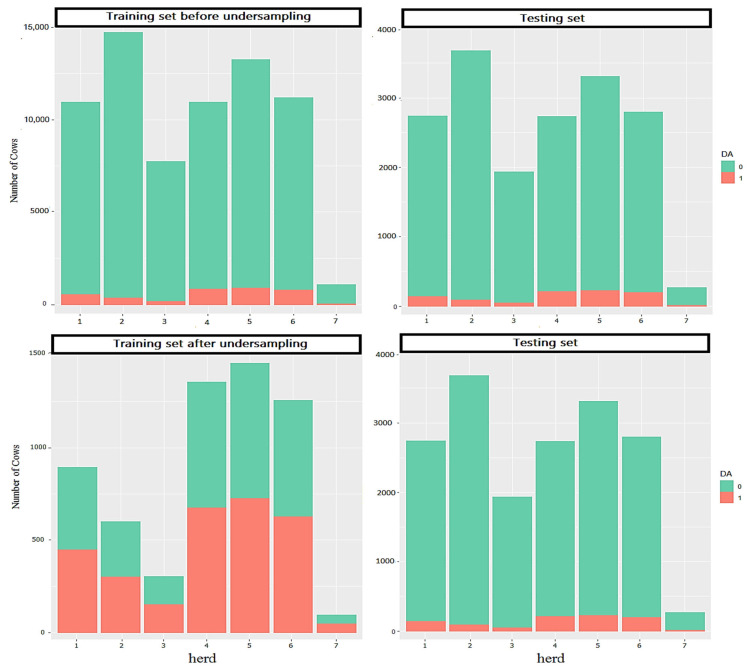
Distribution of displaced abomasum cases of dairy cattle across herds in the training set (before and after under-sampling) and testing set.

**Figure 3:**
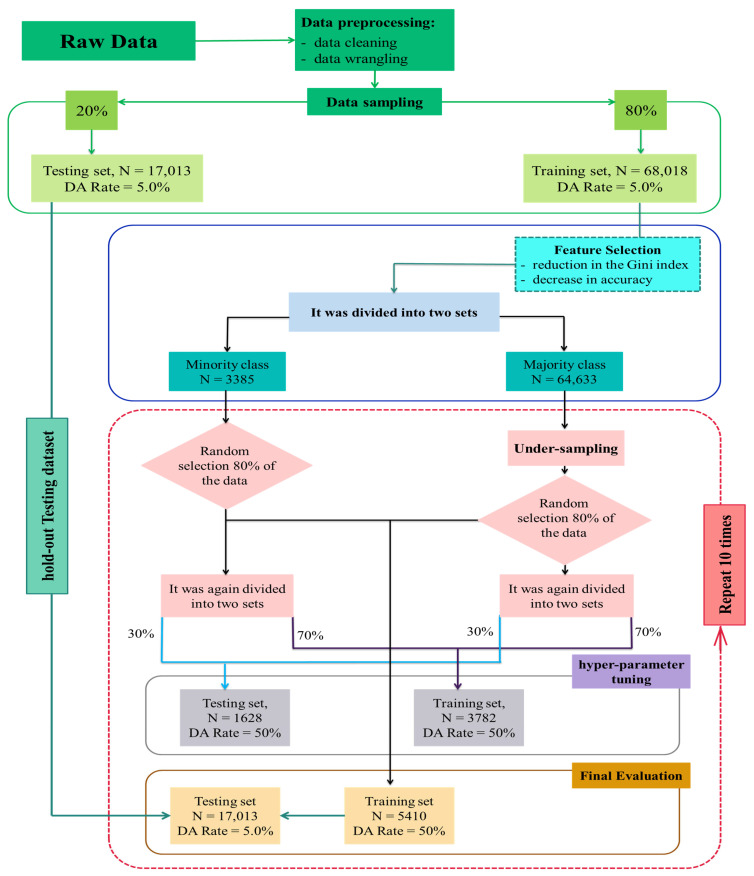
Flowchart of the resampling of the original dataset to generate the balanced set and data used for model tuning. Under-sampling performed via ten random samples from unaffected cows of each herd to the total number of cows with DA without replacement.

The average incidence rate of DA was approximately 5.0% among the herds under study, which indicates a highly class-imbalanced data (Figure 2). Classification algorithms that aim to minimize the error rate do not perform well when dealing with imbalanced data. One of the approaches to deal with this problem is the use of sampling techniques, including down-sampling and up-sampling [38]. In the present study, down-sampling techniques were used to produce a balanced training set. Machine learning algorithms were tuned, trained and tested based on the algorithm explained below. First, the raw dataset (after preprocessing and data wrangling) was divided into two sets. For this division, first the raw dataset in each herd divided into two classes, sick (majority class) and healthy (minority class), and then, each dataset was separately divided into two datasets with a ratio of 80:20. Twenty percent of the data from each class were randomly selected and merged together; this dataset was set aside as the holding test set. The remaining 80% of each class after merging was considered as the training data. Then in the second step, after feature selection by using the training set (described above), sampling techniques were used to generate 10 balanced training data. Therefore, to keep the total number of cows with DA constant, the aforementioned 80% training dataset split (to cows with DA and healthy), and down-sampling was only performed in the majority class within each herd to stratify the data within the herd structure and ensure that each herd contributes to the training set equally. Hence, ten random samples were taken from unaffected cows of the training set without replacing to match the total number of cows with DA. In the next step, 80% of each training set was selected at random (these ten samples were used for model training). Furthermore, 70% of each balanced training set was selected for training (For hyperparameter tuning) and 30% for testing (to evaluate performance during hyperparameter tuning) purposes of hyperparameter tuning through a nested grid search. Finally, the prediction models were developed using the best-performing hyperparameters on each of the 10 training sets, and was validated on the imbalanced holed-out testing set. Figure 3 represents an overview of the whole process. Model performance metrics (MPMs) were aggregated over the 10 iterations of training and testing. This approach was used to control the prediction variance and enhance model generalizability [35]. All analysis and models were implemented in R (version 4.1.2; [34]).

To evaluate the performance of the models, common MPMs were used. These MPMs include overall accuracy (ACC = (TP + TN)/(TP + FP + TN + FN)), balanced accuracy (BACC = (TPR + TNR)/2), the true positive rate (TPR = TP/(TP + FN)), the true negative rate (TNR = TN/(TN + FP)), precision (PPV = TP/(TP + FP)), the false negative rate (FNR = FN/(FN + TP)) and the false positive rate (FPR = FP/(FP + TN)), where TP is true positive, TN is true negative, FP is false positive, and FN is false negative. In data with highly imbalanced classes, the TPR is more appropriate for measuring the success of any classifier, because the TPR focuses on performance measurement in the positive or minority class, which provides more relevant and appropriate information [39]. The true positive rate (recall) measures the percentage of the positive examples that the model predicted correctly [40]. Because, the TPR only uses values of one column of the confusion matrix, it cancels the changes in the data class distribution, which is the ratio between the positive and negative samples [41]. Model performance metrics that are estimated based on both columns of the confusion matrix will be sensitive to the imbalanced data [42] and they will change as data distributions change [43].

A scalar metric alone might not be an appropriate parameter to measure the predictive performance of a model, especially for nonparametric models [44]. Hence, to measure and evaluate any classifier in this study, in addition to the TPR, the focus was also on F2-measure. Due to the characteristics of imbalanced data, the F-measure was chosen because its emphasis is on the TPR and PPV to calculate an integrated score (i.e., identification of minority class). F2-measure is calculated as the harmonic mean of precision and recall (TPR) and gives a weight to each one [45]:

(1) $$\text{F2-measure}=\;\frac{\;\left(1+\beta^{2}\right)\times \;\mathrm{TP}}{\left(1+\beta^{2}\right)\times \;\mathrm{TP}\;+{\;\beta }^{2}\times \;\mathrm{FN}\;+\mathrm{FP}\;}$$

where β, which indicates that recall is more important than precision, is a weight control parameter and gives more weight to recall. In the F2-measure, a β of 2 is selected to emphasize minority class learning by assigning more weight to the TPR in the performance of the algorithms [39].

## 3. Results

DA is a complex trait and many predisposing factors are involved in its occurrence. This disease usually occurs early in the postpartum period. In the current study, 63% of cases of DA occurred in the first month after calving. The incidence rate of DA is low and leads to a severe imbalance in the data. Hence, it is difficult to achieve accurate prediction. In the present study, down-sampling techniques were applied for the training dataset used to train DA case prediction models.

**Figure 4:**
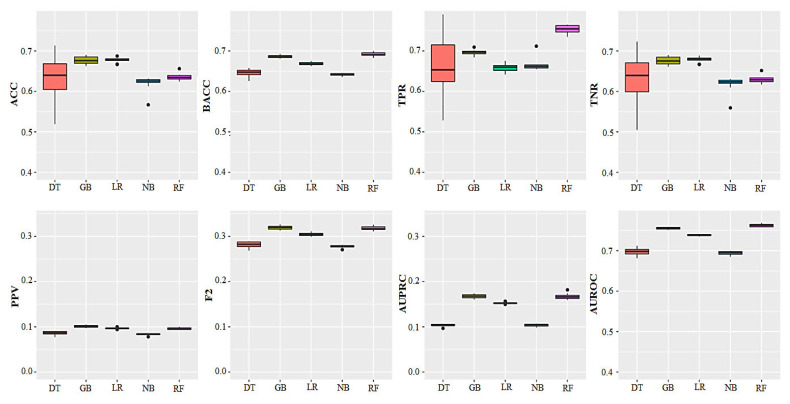
Boxplots of model performance metrics showing prediction variability in different algorithms on the testing set in 10 iterations; DT = Decision Tree, GB = Gradient Boosting (Machines), LR = Logistic Regression, NB = Naïve Bayes, RF = Random Forest, ACC = overall accuracy, BACC = balanced ACC, TPR = true positive rate, TNR = true negative rate, AUROC = Area Under the ROC, PPV = precision, F2 = F2-measure and AUPRC = Area Under the Precision–Recall Curve.

**Figure 5:**
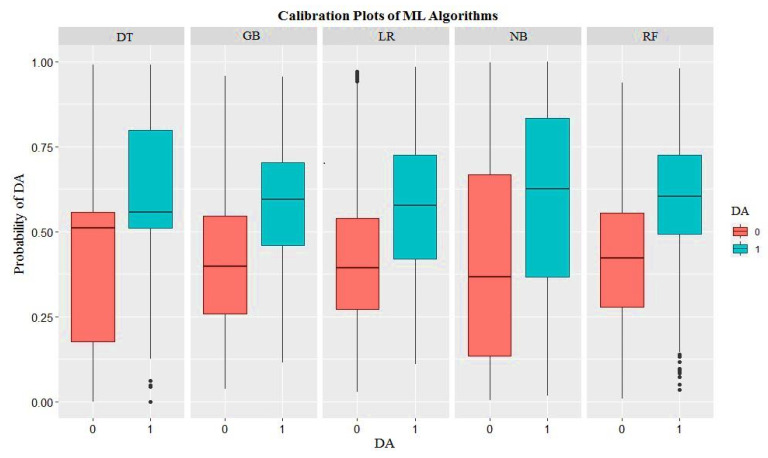
Calibration plots of different machine learning models: Decision Tree (DT), Gradient Boosting (Machine) (GB), Logistic Regression (LR), Naïve Bayes (NB) and Random Forest (RF).

The performance of the different machine learning algorithms used to predict cases of DA using a highly imbalanced dataset (hold-out testing dataset) is shown in Table 4 and Figure 4. Also, the calibration, which indicates the degree to which the predicted risk matches the actual risk, was shown using calibration plots (Figure 5). The classification accuracy of predictive models ranged from 62% to 69%. In terms of ACC, LR and GBM outperformed other machine learning algorithms at 0.68 and 0.69, respectively. The greatest value of F2-measure was achieved with GBM and RF, whereas NB had the lowest (0.33 vs. 0.27). In terms of the TPR, RF outperformed other machine learning algorithms at 0.75, whereas LR was the lowest. Considering the PPV, it was GBM that outperformed other methods at 0.10. PPVs were low for all algorithms and varied between 0.07 (NB) and 0.10 (GBM) across algorithms. The greatest value of AUPRC and AUROC was achieved with GBM (0.17) and RF (0.76), respectively.

## 4. Discussion

The results of the present study demonstrate that considering the PPV, it was GBM that outperformed other methods at 0.10. Implemented models’ low PPVs were expected, because the PPV is a function of the trait incidence rate, and a lower incidence leads to a lower PPV. When predicting a trait with a low incidence rate, a model with a good TPR and TNR can have a low PPV. Although the PPV is calculated directly from TP and FP, it is a function of the incidence rate of the trait under study [46]. In other studies, PPVs for mastitis and lameness are 0.01 and 0.10, respectively [47,48], which is lower than the value reported here. This represents a direct relationship between the PPV and the frequency of trait under investigation [49]. Although GBM and LR (0.69 and 0.68, respectively) performed well in terms of accuracy, accuracy is not a good measure to evaluate the performance of prediction models when dealing with imbalanced data, because it assigns more weight for the class with a high frequency than the rare classes [50]. For this reason, balanced accuracy was also estimated, which weighs the two classes equally and is believed to be more plausible than using accuracy alone in situations of using highly unbalanced data [51]. The difference between the two criteria was very small that indicates that a classifier performs equally well on either class [52]. Table 5 provides a sample of recent studies that attempted to predict health traits in dairy cattle using ML.

In terms of AUROC, RF and GBM also outperformed other machine learning algorithms at 0.76 and 0.75, respectively; AUROC is the appropriate measure of a classifier’s ability and reflects the power of positive prediction probability ranking. Of course, in low incidence rate cases, especially when the frequency of the minority class is lower than 5%, the Area Under the Precision–Recall Curve (AUPRC) is preferred because it has a better agreement with the PPV. In this respect, GBM and RF outperformed other machine learning algorithms by 0.17.

According to the characteristics of the imbalanced data, F2-measure and TPR were used as the main metrics to evaluate the predictive performance of models. In this regard, the predictive performance of the ML algorithms used in the present study, based on F2-measure, varied from 0.27 (NB) to 0.32 (RF and GBM). RF and GBM had the highest F2-measure at 0.32 followed by LR (0.30). Considering the TPR, it was RF that outperformed other methods (0.75). GBM had the highest TPR (0.70) after RF, whereas LR had the lowest performance (0.65). A high TPR, especially when analyzing data related to a disease, is very important for a classifier because in medical data, information is stored in the minority class data and the TPR also measures the performance of a classifier on the minority class [39].

These findings showed the ability of ML algorithms to predict cases of DA. Considering all metrics, the results show that both GBM and RF can have success in predicting DA cases. Other models considered in this study showed similar MPMs. The better performance of GBM and RF can be attributed to the ability of the ensemble approach to form a high-performance predictor based on the training set of collaborative predictors [57]. Previous studies have shown that correlation between predictors can be handled well by these models [58,59]. Random selection of a subset of features to generate any of those classifiers can eliminate the correlation between features. Of course, the method of construction and internal evaluation of both of these algorithms are different, although both are tree-based methods [60].

Although better performance metrics of RF and GBM were statistically significant, results indicate a limited improvement. So that, a large proportion of cows are still misclassified (i.e., low PPV). This will prove the fact that prediction of DA is extremely difficult and even the best algorithm in the current study still provides modest accuracy. The low performance of DA prediction models used in this study was expected because in addition to having a highly imbalanced dataset, information on the mechanism(s) of why DA occurs [61,62] and the coordinated action of many predisposing factors involved in its occurrence [15,16] are unknown to us. Among these factors, weather [20,21], environment and management factors such as nutrition and metabolism [63,64], body score condition [18], energy metabolism markers such as β-hydroxybutyric [3,65] and minerals such as calcium levels in serum [65] can be mentioned but were not available to us in the current study. Having a complete set of factors affecting the occurrence of disease can lead to a better performance of prediction models.

However, to the best of the authors’ knowledge, this is the first study that used predictive models, phenotypic data, and sires’ EBVs to identify cows prone to DA and proved the ability of machine learning models in predicting whether a cow is at high risk of DA during its lactation period. Therefore, it can be considered as a decision support tool to help farm managers in monitoring susceptible cows. Identifying cows prone to metabolic disorders such as DA can lead to better cow health management, and provides farmers with the opportunity to modify diet and farm management strategies to prevent disorders a priori [66]. Susceptible cows usually have a poor metabolic status [12]; a metabolic status can be improved by feeding diets with a higher energy content [67] or by reducing the dry period length. If a cow is prone to metabolic disorders (such as DA) but has a good body condition score, limiting energy supply can lead to reduced body fat storage and a reduced risk of metabolic disorders in the next lactation period [68]. So, utilizing the approach introduced in the current study can aid farmers as an alerting system in order to take preventative actions on cows susceptible to these abnormalities.

According to previous reports, displaced abomasum is a costly disease in all parities [69]. The cost per case of DA has been reported from 450 USD per case [70] to more than 700 USD [8]. As an example of how this model can work effectively in practice, suppose the incidence of DA in a herd of 1000 cows is 5.5%, so 55 cows per year will be affected with DA. Using the approach introduced in this study, it is possible to correctly identify 10% of cows at high risk of DA a priori (PPV 10%, Table 4), which is equivalent to six cows in this hypothetical scenario. As a result, assuming an effective preventive intervention practice is available on time, using this algorithm, 2700 to 4200 USD of future financial losses can be prevented, which translates to higher profits for farmers. This approach can be adapted for other dairy cattle health and welfare issues as well to enhance profitability.

## 5. Conclusions

In the present study, machine learning algorithms were used for initial DA prediction. Among the algorithms considered in this study, RF and GBM were significantly better in terms of F2-measure (0.32) and TPR (0.75 and 0.70, respectively). Other models considered in this study showed similar MPMs. Although, prediction of DA is quite challenging as DA is a complex trait and its low occurrence rate causes severe data imbalance. The primary strength of this study lies in demonstrating the effectiveness of our custom-designed under-sampling and cross-validation algorithm in predicting highly imbalanced outcomes. Suboptimal performance metrics could also be due to the fact that some important DA risk factors were not available to us as predictors, which is expected to be improved with a comprehensive set of DA risk factors as predictor features in the dataset and more advanced predictive models. It is worth mentioning that this is the first study to use predictive models, phenotypic data, and sires’ EBVs to identify cows prone to DA and proves the ability of machine learning models in predicting whether a cow is at high risk of DA during its lactation period.

## Figures and Tables

**Table 1:** Number of excluded records and reasons for excluding them for each parity.

Service Sire PTAs ^1^	Parity > 6	AFC	PL	Milk	Protein	Fat	DPL	Calf Weight	No Milk Record ^2^
1978	2055	244	117	124	1290	503	533	816	582

^1^ Removal due to lack of Service Sire PTAs; ^2^ primiparous cows with DA and without a monthly record of milk.

**Table 2:** Description of features (explanatory variables) used for predicting displaced abomasum in dairy cows.

No.	Features	Type	Level	Minimum (%) ^3^	Maximum (%) ^4^	Mean	SD
**Herd and cow information ^1^**							
**1**	**Herd**	**Nominal**	**7**	**1 (15%)**	**7 (7%)**	**-**	**-**
**2**	**Calving years**	**Nominal**	**11**	**2010 (3%)**	**2020 (16%)**	**-**	**-**
**3**	**Parity**	**Nominal**	**6**	**1 (34%)**	**6 (5%)**	**-**	**-**
**4**	**month of the milk record**	**Nominal**	**12**	**1 (8.6%)**	**12 (9.4%)**	**-**	**-**
5	Calving season	Nominal	4	1 (22%)	4 (24%)	-	-
**6**	**Milk yield**	**Numeric**	**-**	**3.0**	**78.0**	**40.9**	**12.3**
**7**	**Milk fat yield**	**Numeric**	**-**	**0.29**	**7.2**	**3.7**	**1.1**
**8**	**Milk protein yield**	**Numeric**	**-**	**1.1**	**5.1**	**3.0**	**0.4**
**9**	**Age at first calving**	**Numeric**	**-**	**578**	**1153**	**720**	**55**
**10**	**Pregnancy**	**Numeric**	**-**	**240.0**	**298.0**	**275.0**	**5.1**
**11**	**Calf weight at birth**	**Numeric**	**-**	**20**	**53**	**40.0**	**6.2**
**12**	**Dry period length**	**Numeric**	**-**	**0**	**160**	**41.1**	**33.2**
**13**	**Ketosis**	**Binary**	**2**	**0 (98%)**	**1 (2%)**	**-**	**-**
14	Mastitis	Binary	2	0 (83%)	1 (17%)	-	-
15	Metritis	Binary	2	0 (86%)	1 (14%)	-	-
16	Retained placenta	Binary	2	0 (92%)	1 (8%)	-	-
17	Milk fever	Binary	2	0 (98%)	1 (2%)	-	-
18	Dystocia	Binary	2	1 (96%)	2 (4%)	-	-
19	Twinning	Binary	2	1 (97%)	2 (3%)	-	-
20	Calf sex	Binary	2	1 (51%)	2 (49%)	-	-
**Service Sire PTAs ^2^**							
**21**	**PTA-DA**	**Numeric**	**-**	**−2.9**	**1.4**	**−** **0** **.02**	**0.5**
**22**	**PTA-SSB**	**Numeric**	**-**	**3.8**	**10.2**	**6.1**	**0.8**
**23**	**PTA-DSB**	**Numeric**	**-**	**2.6**	**16.7**	**6.5**	**1.7**
**24**	**PTA-SCE**	**Numeric**	**-**	**1.0**	**6.6**	**2.2**	**0.5**
**25**	**PTA-DCE**	**Numeric**	**-**	**1.0**	**5.5**	**2.6**	**0.6**
**26**	**PTA-DPR**	**Numeric**	**-**	**−7.5**	**6.9**	**−0.7**	**2.1**
**27**	**PTA-PL**	**Numeric**	**-**	**−7.6**	**6.3**	**−0.2**	**0.2**
**28**	**PTA-Ket**	**Numeric**	**-**	**−3.7**	**3.0**	**−0.1**	**2.4**
**29**	**PTA-fat%**	**Numeric**	**-**	**−0.3**	**0.4**	**−0.07**	**0.1**
30	PTA-SCS	Numeric	-	2.4	3.6	3.0	1.0
31	PTA-protein%	Numeric	-	−0.2	0.2	−0.3	0.04

^1^ Parity consisted of 1 (34%), 2 (26%), 3 (18%), 4 (11%), 5 (6%) and 6 (5%). For all primiparous cows, the length of the dry period was considered zero. For all records related to disease and reproduction, 0 was no and 1 was yes. For calf gender, 1 was considered a female calf and 2 was considered a male calf. ^2^ PTA of displaced abomasum, sire stillbirth, daughter stillbirth, sire calving ease, daughter calving ease, the daughter pregnancy rate and productive life, somatic cell score, Ketosis, milk protein percentage and milk fat percentage. ^3^ and ^4^ Proportions and frequencies for each level of categorical and binary features were shown in parentheses. Features selected during feature selection stage are shown in bold face.

**Table 3:** Frequency of different levels of nominal features used in the forecasting.

	Level	1	2	3	4	5	6	7	8	9	10	11	12
Features													
Herd		15%	20%	10%	15%	18%	15%	7%	-	-	-	-	-
Calving year		3%	2%	3%	4%	5%	7%	12%	14%	15%	17%	16%	-
month of the milk record		8.6%	7.3%	7.7%	7.5%	7.5%	7.0%	8.0%	10.0%	9.0%	9.0%	8.0%	9.4%
Calving season		22%	27%	27%	24%	-	-	-	-	-	-	-	-
Parity		34%	26%	18%	11%	6%	5%	-	-	-	-	-	-

**Table 4:** Model performance metrics for algorithms used in prediction of the incidence of DA in dairy cows for the testing set.

Algorithm ^1^	ACC ^2^	Balanced ACC	PPV ^3^	TPR ^4^	TNR ^5^	F2	AUPRC	AUROC
Testing dataset								
LR	0.68 (±0.00) ^a^	0.66 (±0.00) ^b^	0.09 (±0.00) ^b^	0.65 (±0.01) ^b^	0.68 (±0.00) ^b^	0.30 (±0.00) ^b^	0.15 (±0.00) ^b^	0.73 (±0.00) ^c^
NB	0.62 (±0.02) ^b^	0.64 (±0.00) ^c^	0.07 (±0.00) ^c^	0.66 (±0.01) ^b^	0.62 (±0.02) ^b^	0.27 (±0.00) ^c^	0.10 (±0.00) ^c^	0.69 (±0.00) ^d^
DT	0.63 (±0.05) ^b^	0.64 (±0.00) ^c^	0.08 (±0.00) ^c^	0.66 (±0.07) ^b^	0.63 (±0.06) ^b^	0.28 (±0.00) ^c^	0.10 (±0.00) ^c^	0.70 (±0.00) ^d^
RF	0.64 (±0.01) ^b^	0.69 (±0.00) ^a^	0.09 (±0.00) ^b^	**0.75 (±0.01) ^a^**	0.63 (±0.01) ^a^	**0.32 (±0.00) ^a^**	0.17 (±0.00) ^a^	**0.76 (±0.00) ^a^**
GBM	**0.69 (±0.01) ^a^**	**0.68 (±0.00) ^b^**	**0.10 (±0.00) ^a^**	**0.70 (±0.00) ^b^**	**0.67 (±0.01) ^a^**	**0.32 (±0.00) ^a^**	**0.17 (±0.00) ^a^**	**0.75 (±0.00) ^b^**
Training dataset								
LR	0.67 (±0.00)	0.67 (±0.00)	0.68 (±0.00)	0.67 (±0.00)	0.68 (±0.00)	0.67 (±0.00)	0.73 (±0.00)	0.74 (±0.00)
NB	0.64 (±0.00)	0.64 (±0.00)	0.63 (±0.01)	0.67 (±0.01)	0.61 (±0.02)	0.66 (±0.01)	0.67 (±0.00)	0.69 (±0.00)
DT	0.68 (±0.01)	0.68 (±0.01)	0.67 (±0.01)	0.70 (±0.06)	0.66 (±0.05)	0.69 (±0.05)	0.72 (±0.03)	0.74 (±0.01)
RF	0.68 (±0.00)	0.68 (±0.00)	0.66 (±0.00)	0.74 (±0.01)	0.62 (±0.01)	0.72 (±0.01)	0.73 (±0.00)	0.75 (±0.00)
GBM	0.72 (±0.01)	0.72 (±0.01)	0.72 (±0.00)	0.74 (±0.00)	0.71 (±0.01)	0.73 (±0.00)	0.80 (±0.00)	0.80 (±0.01)

^1^ LR: Logistic Regression, NB: Naïve Bayes, DT: Decision Tree, RF: Random Forest, GBM: Gradient Boosting Machines; ^2^ accuracy; ^3^ precision or positive predictive value (PPV), ^4^ true positive rate (TPR); ^5^ true negative rate (TNR). According to the Tukey-HSD multiple comparison test, the values in each column are significantly different (*p* < 0.05), which are also shown with different superscript. The bold values in the table indicate the best performance.

**Table 5:** The accuracy of recent studies in predicting health traits in dairy cattle.

Number of Cows	Predicted Trait	Accuracy	Algorithm with Best Performance	Country	Year
2535	Lameness	0.83	Naïve Bayes	Australia	2021 [28]
1000	Neosporosis	0.82	Neural Network	Colombia	2025 [29]
882	Ketosis	0.72	Logistic Regression	Poland	2021 [53]
363,945	Retained Placenta	0.78	XGBoost and Random Forest	Iran	2025 [31]
14,755	Udder Health Status	≥0.75	Neural Network and Random Forest	Italy	2021 [54]
1909	Health Status	0.95	Neural Network	New Zealand	2021 [55]
297,004	Subclinical Mastitis	≥0.83	GBM and Deep Learning	New Zealand	2019 [56]

## Data Availability

The datasets presented in this article are not readily available due to restrictions (commercial restrictions, ethical reasons and privacy). General information about the datasets presented in this study is included in the article. Further inquiries can be directed to the corresponding author (shahinfar@uwalumni.com).
